# Pediatric Ramsay Hunt Syndrome: Analysis of Three Cases

**DOI:** 10.1155/2015/971249

**Published:** 2015-09-07

**Authors:** İmran Aydoğdu, Enes Ataç, Ziya Saltürk, Yavuz Atar, Erdi Özdemir, Yavuz Uyar, Ahmet Arslanoğlu, Güler Berkiten

**Affiliations:** Okmeydanı Training and Research Hospital ENT Clinic, Darülaceze Caddesi, Şişli, 34030 Istanbul, Turkey

## Abstract

Ramsay Hunt syndrome (RHS) is a disorder characterized by herpetic eruptions on the auricle, facial paralysis, and vestibulocochlear dysfunction and is attributed to varicella zoster virus (VZV) infection in the geniculate ganglion. Although it is a common cause of acute peripheral facial paralysis, children are not usually affected. The diagnosis is based on history and physical findings. Treatment of RHS uses a combination of high-dose corticosteroids and acyclovir. This paper presents three cases diagnosed as RHS in the pediatric age group in association with the literature review. The aim of this paper is to emphasize the importance of careful examination and early initiation of therapy in suspected cases of RHS.

## 1. Introduction

Ramsay Hunt syndrome (RHS) is a rare condition characterized by peripheral facial paralysis, an auricular skin rash, and cochleovestibular symptoms. RHS develops following the reactivation of latent varicella zoster virus (VZV) in the geniculate ganglion, thus affecting the seventh and eighth cranial nerves [1, 2]. In patients with chickenpox, the virus enters the ganglion through sensory branches of the facial nerve located in the ear and tongue. Only 10% of cases of facial paralysis in pediatric populations are associated with RHS. Although RHS is rare in children, it is the second most common cause of facial paralysis (after Bell's palsy) in children with nontraumatic peripheral facial paralysis [3]. The reported incidence is 2.7/100,000 children [4]. Diagnosis is based on history-taking and physical findings. Here, we present three cases of RHS in children and a review of the literature.

## 2. Case 1

**Figure 1:**
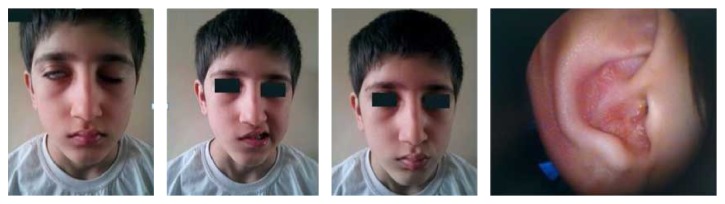
An 11-year-old male with right facial paralysis and vesicular lesions in the right auricula.

An 11-year-old male complained of fever, a sore throat, and ear pain 1 week in duration; he was diagnosed with acute otitis media and administered oral antibiotics. One week later, the patient was unable to fully close his right eye and the right nasolabial groove was absent on a follow-up neurological examination. He was diagnosed with Bell's palsy and oral treatment with methylprednisolone (1 mg/kg/day) was commenced. On the second day of treatment, he was referred to our clinic because painful blisters filled with water had developed in his right ear. The patient had Grade 4 House-Brackmann (HB) facial paralysis (Figure 1). Laboratory findings (complete blood count, erythrocyte sedimentation rate, and blood data) were within normal limits. The patient had a bilateral type A tympanogram, and audiography yielded pure-tone thresholds of 6 and 5 dB in the right ear and left ear, respectively. As the characteristic rash had developed in a typical location and the patient's facial paralysis was peripheral in nature, he was diagnosed with RHS and a 5-day course of intravenous acyclovir (10 mg/kg/day) was commenced. The patient was followed-up through office visits; the ear lesions disappeared within 3 weeks, and the facial paralysis resolved completely by the end of 6 weeks.

## 3. Case 2

**Figure 2:**
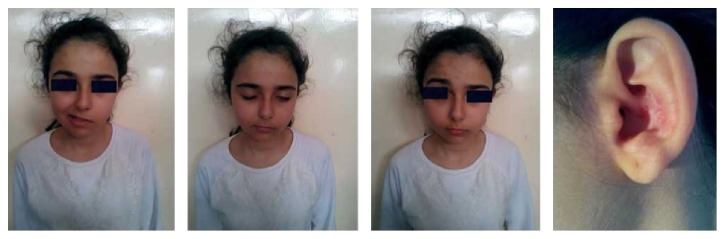
A 12-year-old female with left facial paralysis and a vesicular lesion in the left auricula.

A 12-year-old female was referred to our clinic because of facial asymmetry. She complained of a sore throat and cough 3 days in duration. She also had pain in her left ear, a vesicular rash in the auricular region, and facial asymmetry. On examination, she exhibited facial paralysis but lacked cochleovestibular symptoms such as hearing loss, vertigo, vomiting, and tinnitus. She had facial palsy of HB Grade 3 (Figure 2). Laboratory data (complete blood count, erythrocyte sedimentation rate, and blood data) were within normal limits. She had a bilateral type A tympanogram, and audiography yielded pure-tone thresholds of 10 and 12 dB for the right ear and left ear, respectively. The patient was hospitalized, and a 5-day course of acyclovir (800 mg/day) and a 14-day course of methylprednisolone (2 mg/kg/day) were commenced. The corticosteroid was reduced to 1 mg/kg/day after 1 week. The patient's facial paralysis was resolved completely by the end of the first week.

## 4. Case 3

**Figure 3:**
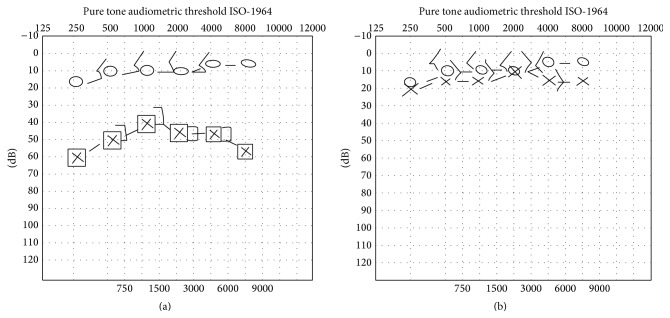
Audiographic results before and after treatment. (a) Before treatment. (b) After treatment.

A 12-year-old male patient was admitted with initial complaints of left ear pain, vertigo, and vomiting; he then developed typical vesicular rashes in the auricle and external auditory canal. Left peripheral facial nerve paralysis (HB Grade 3) developed 2 days after admission. He also exhibited sensorineural hearing loss; the pure tone audiometric threshold was 40 dB in the left ear (Figure 3). No sign of central nervous system involvement was evident on follow-up visits. He was hospitalized with a diagnosis of RHS, and both antiviral therapy and corticosteroid therapy were commenced. An antiemetic and a sedative were also prescribed to control the vestibular symptoms. Five days later, the pain had ceased. The vesicular rashes in the auricle and external auditory canal and the vestibular symptoms began to improve on day 6 and had completely disappeared by day 10. An average 20 dB of improvement in the patient's sensorineural hearing loss was evident upon audiometric examination of the left ear on day 10. On day 20, facial paralysis was still present, but the extent thereof had declined. The patient's facial paralysis, hearing loss, and tinnitus resolved within the first month of follow-up visits.

## 5. Discussion

RHS (also known as herpes zoster oticus) is characterized by facial paralysis, ear pain, and erythematous vesicular lesions in the ear and oral mucosa [1, 2]. These characteristic vesicles usually develop on the external auditory canal, pinna, and (rarely) on the anterior tonsillar folds [5]. RHS, which develops in 1% of patients with a herpes zoster infection, was first described by Ramsay Hunt in 1907 [6]. Although the syndrome is common in adults and older children, it is rare (and much milder) in young children. RHS caused by varicella zoster reactivation is responsible for 16.7% of cases of facial paralysis in pediatric populations [7].

RHS commences with the classical prodromal symptoms of pain, fever, and fatigue, 1–3 days in duration. Next, herpetic vesicles begin to develop in the external auditory canal, tympanic membrane, and/or the anterior two-thirds of the tongue. Facial paralysis usually develops within 1–2 weeks after the rash appears [8, 9]. The condition is sometimes accompanied by symptoms of eighth nerve involvement (nausea, vomiting, vertigo, nystagmus, tinnitus, and hearing loss). Hearing loss develops in 24% of affected children [10]. Two of our patients developed vesicular lesions before the onset of facial paralysis; the reverse was true of the other patient. One patient exhibited eighth nerve involvement. The cochleovestibular symptoms associated with such involvement (tinnitus, hearing impairment, and vertigo) are less common in children than adults [5, 11]. In one of our cases, the cochleovestibular symptoms were more prominent than the facial paralysis. VZV reactivation is thought to occur in both the spiral and vestibular ganglia of such cases.

RHS is definitively diagnosed by measuring serum anti-VZV IgG and IgM antibody titers using ELISAs [12]. However, serological investigations were not required in our three cases because characteristic findings were present.

Early commencement of antiviral and high-dose steroid therapy has become the standard treatment for patients diagnosed with RHS at many centers. Antiviral agents inhibit viral replication, restrict new lesion formation, and afford rapid lesional healing [3]. Steroids reduce edema and pain by countering inflammation in peripheral neurons. Commencement of an antiviral agent within the first 72 h is important if treatment is to be effective [10, 13]. Combined antiviral and steroid treatment is more effective than steroids alone [14]. Such combined treatment markedly reduces acute pain, accelerates recovery, and improves the patient's quality of life [15]. According to Kinishi et al. [16], the complete recovery rates for RHS patients were 62% when (only) steroid treatment was commenced within 1 week, but 90% in those treated with both a steroid and acyclovir. When combined antiviral and steroid treatment commenced at 3–5 days, at 3–7 days, and on day 7, the recovery rates were 75, 48, and 30%, respectively [17]. Audiovestibular symptoms, late commencement of treatment, and advanced facial asymmetry are indicative of a poor prognosis [8]. We commenced early acyclovir and methylprednisolone therapy in all patients, and we applied an eye gel and treated eye closure to prevent the development of dry eye. Antiemetic and sedative therapy were prescribed in the case with eighth nerve involvement. All patients recovered without sequelae.

The symptoms and signs of RHS are more severe than those of Bell's palsy, and the prognosis is poorer [18]. Hato et al. [7] retrospectively evaluated 52 RHS patients; the remission rates were 78.6% in pediatric patients (aged <16 years) and 49% in adult patients. Cranial neuropathies were rarer in the pediatric than the adult population.

In conclusion, two cases experienced ear pain as the first symptom, whereas one had initial cochleovestibular symptoms. No patient had a history of immunosuppression. The patients were diagnosed 2 days, 3 days, and 1 week after disease onset, and treatment commenced immediately. The patient whose treatment commenced on day 2 recovered within 1 week. The patient with cochleovestibular symptoms whose treatment commenced on day 3 improved by the end of 1 month. He thus recovered slowly despite early treatment initiation. This is because cochleovestibular symptoms are associated with a poor RHS prognosis. The patient who responded most slowly to treatment was the patient for whom treatment commenced 1 week after symptom development. We believe that early initiation of antiviral/corticosteroid combination therapy is important for the treatment of RHS. Early prescription of acyclovir increases the survival rate of the facial nerve.
